# Feasibility of Vibroacoustic Sensing for Detection of Peritoneal Entry During Laparoscopic Access: A Pilot Study in a Human Body Donor

**DOI:** 10.3390/diagnostics16121780

**Published:** 2026-06-09

**Authors:** Moritz Spiller, Robin Urrutia, Nazila Esmaeili, Axel Boese, Thomas Neumuth, Alfredo Illanes, Salmai Turial

**Affiliations:** 1 Innovation Center Computer Assisted Surgery (ICCAS), University of Leipzig, 04103 Leipzig, Germany; moritzspiller@gmail.com (M.S.);; 2 Faculty of Computer Science and Research Campus STIMULATE, Otto-von-Guericke University, Universitätsplatz 2, 39106 Magdeburg, Germany; robin.urrutia@ovgu.de (R.U.); alfredo.illanes@ovgu.de (A.I.); 3 Center for Digital Surgery, Department of General, Visceral, and Pediatric Surgery, University Medical Center Goettingen, Robert-Koch-Straße 40, 37075 Goettingen, Germany; nazila.esmaeili@med.uni-goettingen.de; 4 INKA Innovation Laboratory for Image Guided Therapy, Otto-von-Guericke University, Universitätsplatz 2, 39106 Magdeburg, Germany; 5 Clinic and Polyclinic for Pediatric Surgery, Mainz University Medical Center, Langenbeckstr. 1, 55131 Mainz, Germany; salmai.turial@unimedizin-mainz.de

**Keywords:** capnoperitoneum, guidance systems, laparoscopy, laparoscopic access, minimally invasive surgery, surgical support, vibroacoustics, SURAG research kit

## Abstract

**Background/Objectives:** Establishing laparoscopic access remains a critical and complication-prone step in minimally invasive surgery. Previous work has shown that proximal vibroacoustic sensing can identify peritoneal puncture events in porcine cadavers. The present pilot study evaluated whether these findings translate to human anatomy under controlled, ex vivo conditions. **Methods:** A vibroacoustic sensing prototype was proximally attached to a standard Veress needle during 14 insertions into a fresh human body donor (within 48 h post-mortem). An endoscope was introduced laterally to provide visual ground truth of peritoneal entry. Vibroacoustic signals were recorded at the proximal end of the instrument. Time–frequency analyses, transient excitation detection, and statistical comparisons were performed to assess whether (1) peritoneal puncture can be identified in the vibroacoustic signal, (2) signal phases and dynamics correspond to those previously observed in porcine cadavers, and (3) peritoneal punctures can be statistically differentiated from non-peritoneal events. **Results:** All 14 peritoneal punctures were identifiable in the vibroacoustic signal under the experimental conditions. Characteristic signal phases previously described in porcine tissue, including transient excitation associated with cavity entry, were consistently reproduced with comparable temporal and spectral profiles. Statistical analyses demonstrated group-level differences between peritoneal and non-peritoneal events, and the peritoneal puncture was the highest-energy event of its insertion in 13 of 14 cases (92.9%). **Conclusions:** Under the controlled ex vivo conditions of this single-donor pilot study, vibroacoustic sensing was feasible for identifying peritoneal puncture in human tissue and reproduced signal dynamics observed in porcine models. To our knowledge, this is the first demonstration of the proximal vibroacoustic sensing concept on a human body donor and the first cross-species replication of the previously reported puncture phase structure, establishing an important translational stepping stone between animal cadaver studies and in vivo investigations. The study demonstrates feasibility rather than clinical reliability: the single-donor design and the retrospective annotation framework limit generalizability. Prospective validation in living patients, across multiple subjects and operators, is required before clinical deployment.

## 1. Introduction

Minimally invasive surgery (MIS) has transformed surgical practice by offering patients shorter recovery times, less postoperative pain, and reduced blood loss compared to traditional open surgery [1]. Establishing laparoscopic access nevertheless remains the most critical and hazardous step of the procedure, accounting for 30–50% of all laparoscopic complications [2,3,4,5,6]. Up to 50% of intestinal and vascular injuries remain undetected intraoperatively, often leading to sepsis and mortality rates of 5–15% [7,8,9], and underreporting suggests that the true incidence is higher still [9,10,11,12,13].

The Veress needle, introduced in the 1930s, remains the most widely used closed-entry tool because of its simplicity, low cost, and central role in surgical training. Its safety, however, depends almost entirely on the surgeon’s tactile perception and on the auditory cue of the spring mechanism during abdominal-wall penetration—an inherently subjective and error-prone technique. Even experienced operators report uncertainty: 36% feel unsafe using the Veress needle, and 55% state that its spring mechanism does not clearly indicate peritoneal entry [14,15].

The principal alternative is the open (Hasson) technique, in which a mini-laparotomy is created under direct vision before trocar insertion. Systematic reviews and meta-analyses report no statistically significant difference in major vascular or visceral injury between the two approaches when performed by experienced surgeons [16,17], although pooled data consistently show higher rates of failed entry, preperitoneal insufflation, and omental injury with the Veress needle [17,18]. Open access is therefore considered particularly advantageous in obese patients or those with prior abdominal surgery, where blind insertion is more hazardous due to increased abdominal-wall depth and distorted anatomy [19]. Numerous device-based alternatives have been proposed, but despite decades of innovation safe laparoscopic access cannot be guaranteed [7,10,20,21]: optical trocars provide real-time visualization yet remain costly, bulky, and complication-prone (the FDA has documented 79 severe injuries despite their use) [22,23,24,25]; modified Veress needles such as LaparoLight (ConMed) and EpiAccess (EpiEP), and prototypes with integrated cameras or OCT probes, are hindered by high development costs and regulatory hurdles [26,27]; sensor-augmented trocars and needles have demonstrated feasibility only in experimental models [28,29,30]; and mechanical adjuncts such as LevaLap [31,32] or technique modifications using existing tools [7,33] offer only incremental gains and do not eliminate unrecognized intra-abdominal injury. Conventional Veress needles and trocars therefore continue to dominate clinical practice, and their inability to reliably detect peritoneal entry underscores the need for guidance solutions that improve safety while integrating seamlessly into established workflows.

Vibroacoustic sensing of surgical tool–tissue interaction is one such modality. Interactions between the instrument and biological tissues generate characteristic vibroacoustic signals at the interaction site, which propagate naturally through the tool to its proximal end [34]. Acquisition at the proximal end makes the technology non-invasive—no active component is required inside the patient—and allows it to be implemented as an add-on system without structural modification of the surgical instrument. Previous work has demonstrated correlations between vibroacoustic dynamics and insertion force [34] and has shown that distinct spectral and temporal features identify transitions between tissue types and the puncture of major anatomical layers [35]. Specifically for the Veress needle, vibroacoustic sensing has been investigated in ex vivo tissue and animal cadavers [36,37], where the recorded signatures discriminate key insertion phases such as passage through subcutaneous layers, fascia puncture, and peritoneal entry, supporting its feasibility for objective real-time monitoring of needle placement.

The study described in this article is an extension of the work published in [37]. Vibroacoustic signals were acquired using an easily integratable data acquisition system that digitalizes dynamic processes of interventional tools (SURAG Medical GmbH, Leipzig, Germany) during Veress needle insertion into a single human body donor. The acquired signals were post-processed in a retrospective, video-anchored analysis framework to assess whether:

1. The puncture to the peritoneum of the human body donor can be identified in the vibroacoustic signal.
2. The signal phases observed during Veress needle puncture of the peritoneum in porcine cadavers can also be observed in human body donors, especially with respect to signal energies and temporal durations.
3. It is feasible to statistically differentiate the signal characteristics of peritoneum and non-peritoneum punctures.

The study was structured as a translational feasibility (pilot) investigation. Its primary aim is to validate, in human anatomy and under controlled ex vivo conditions, that the vibroacoustic signatures previously reported in porcine cadavers are reproducible. Limitations relevant to clinical translation, in particular the use of a single body donor and the retrospective annotation framework, are explicitly considered in the Discussion (Section 4).

## 2. Materials and Methods

In this study, vibroacoustic signals were obtained by inserting Veress needles into a single human body donor. Post-experiment signal analysis was performed to determine whether peritoneum punctures could be reproducibly identified in vibroacoustic signals. The following section describes in detail the experimental setup and methods used.

### 2.1. Experimental Setup and Data Acquisition

**Figure 1:**
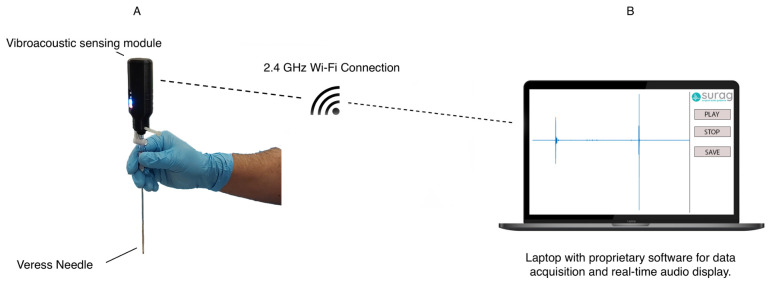
Proximal vibroacoustic sensing prototype. (**A**) Sensing unit mounted to the proximal Luer-lock of the Veress needle. (**B**) Laptop running the acquisition and real-time feedback application. The two devices communicate over a 2.4 GHz Wi-Fi link.

The vibroacoustic sensing prototype used in this study (SURAG Medical GmbH, Leipzig, Germany) was specifically designed for Veress needle interventions and is shown in Figure 1. It consists of a proximal sensing unit and a Lenovo T490 laptop (Lenovo, Hongkong, China) for signal acquisition, storage, and real-time feedback. The sensing unit comprises a custom printed circuit board (PCB) with buttons, LEDs, and a sensing element, mounted on a Raspberry Pi Zero W (Raspberry Pi Foundation, Cambridge, UK). Its housing terminates in a male Luer connector that mates with the female Luer of a 150 mm single-use Veress needle (Applied Medical, Rancho Santa Margarita, CA, USA), so that the needle itself remains unmodified. A proprietary application (SURAG Medical GmbH, Leipzig, Germany) built in Python 3.6 receives the streamed signal over the wireless link and stores it as a WAVE file at a sampling rate of 16 kHz on the laptop’s internal hard disk. During each insertion, the application also provided real-time visual feedback (the time-domain trace of the vibroacoustic signal) and processed acoustic feedback that amplified the dominant needle-tip interactions; these were used for exploratory monitoring and to verify correct operation of the prototype and wireless connection.

**Figure 2:**
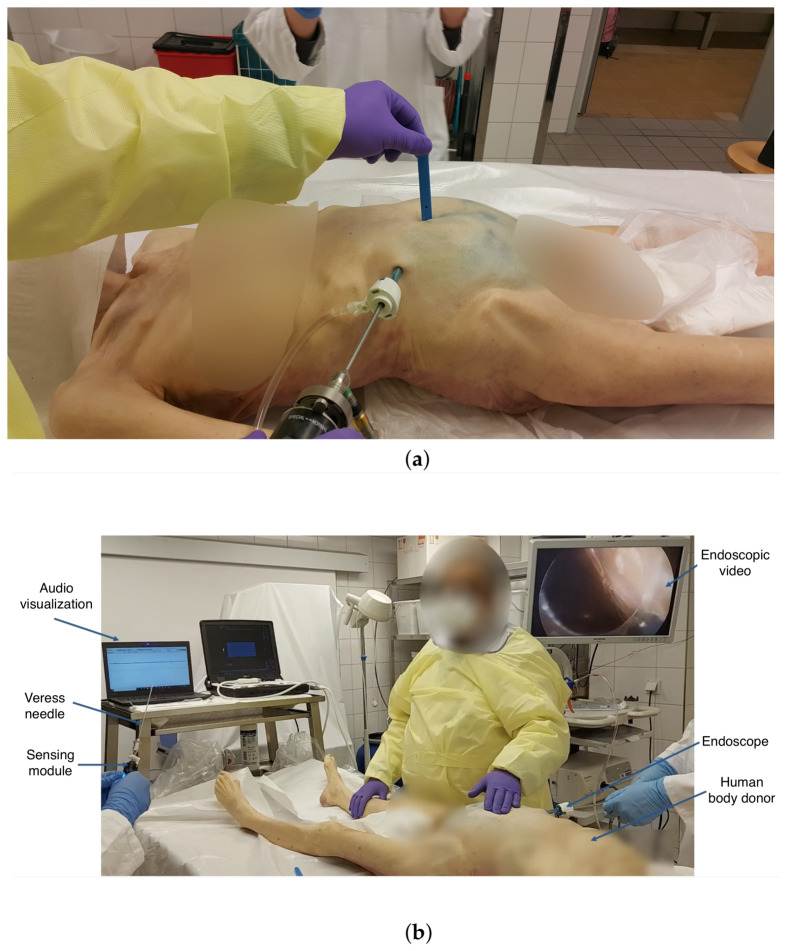

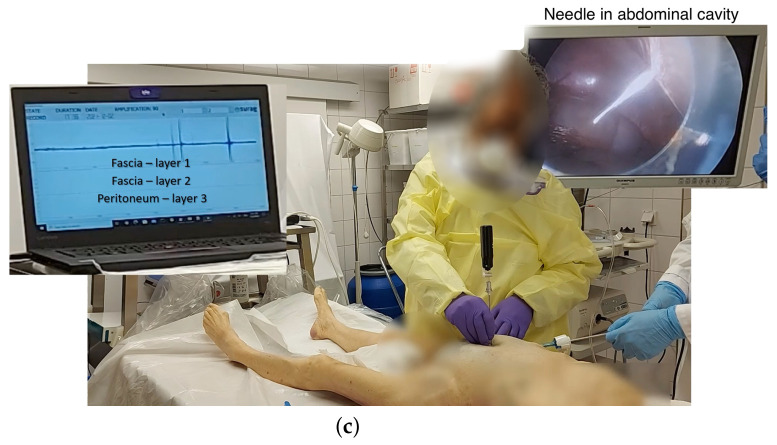
The experimental setup for acquiring vibroacoustic signals from Veress needle insertions into a human body donor. Overall, 14 insertions in a single human body donor were performed using this setup. (**a**) An endoscopic camera was introduced before Veress needle insertion to obtain ground truth. (**b**) The needle was inserted at 90 degrees and the moment that the needle punctures the peritoneum can be visualized in the endoscopic camera image. (**c**) The acquired vibroacoustic signal was displayed as auditory and visual feedback in real-time throughout the insertion process.

Figure 2 illustrates the experimental setup used to acquire audio signals from Veress needle insertions. Vibroacoustic signals were obtained from a single human body donor within 48 h post-mortem. The donor had no documented history of prior abdominal surgery and no visible adhesions or scars in the operative field.

Prior to the Veress needle insertions, a trocar and an endoscope were inserted laterally on the donor’s right side to visually observe the Veress needle’s entry into the peritoneal cavity. Because the cadaver’s abdominal wall had collapsed, slight pressure had to be applied to the abdominal cavity to allow the camera to see. This was performed by an experienced laparoscopic surgeon. The pressure generated was significantly lower than standard clinical pressure (10–12 mmHg). The extent to which this affects the study’s results is discussed in Section 4.

The prototype was mounted proximally on the Luer lock of the Veress needle, and the laptop provided the acquired vibroacoustic signal as acoustic and visual display during the experiment. Visual and acoustic display was chosen for exploratory purposes and to observe whether the devices and wireless connection were functioning correctly. In addition, an observation camera was incorporated into the experimental setup, allowing recording of the entire procedure and indirect access to the peritoneal puncture event by capturing the instant it appeared on the monitor. The recordings of the endoscopic camera, the observation camera, and the prototype were synchronized and stored for subsequent data analysis.

In each experiment, the body donor was placed supine on the operating table. After cleaning the abdominal area, the Veress needle insertion sites were marked in the abdomen, based on commonly recommended primary entry points: the subumbilical region, Lee–Huang point (in the upper midline) and Palmer’s point (left upper quadrant, mid-clavicular line, approximately 3 cm below the costal margin) [38]. The 14 insertions were distributed across these sites as summarized in Table 1. Insertion sites were spatially separated by a minimum of approximately 2 cm, and adjacent sites were not punctured in immediate succession, but the cumulative effect of repeated insertions in a single donor cannot be excluded and is acknowledged in Section 4.

For subsequent data analysis, a strict synchronization protocol was applied. At the beginning of each insertion, three distinct deliberate excitations of the needle were produced. These excitations were clearly audible in both, the vibroacoustic signal and the audio track of the observation video. These three excitations served as synchronization markers, allowing precise alignment of the vibroacoustic signal timeline with the video timeline.

At each marked location, a 5 mm skin incision was made and a Veress needle was slowly inserted at a 90° angle relative to the abdomen, following standard laparoscopic access procedures. The insertions were performed by a single experienced laparoscopic surgeon to minimize operator-related variability; this also constrains generalizability and is noted in Section 4. Before each insertion, the sensing module was attached to the proximal end of a 150 mm single-use Veress needle (Applied Medical, Rancho Santa Margarita, CA, USA), as also described in Section 2.1. To ensure sharpness, the needle was replaced with a new one every five insertions.

### 2.2. Data Analysis

**Figure 3:**
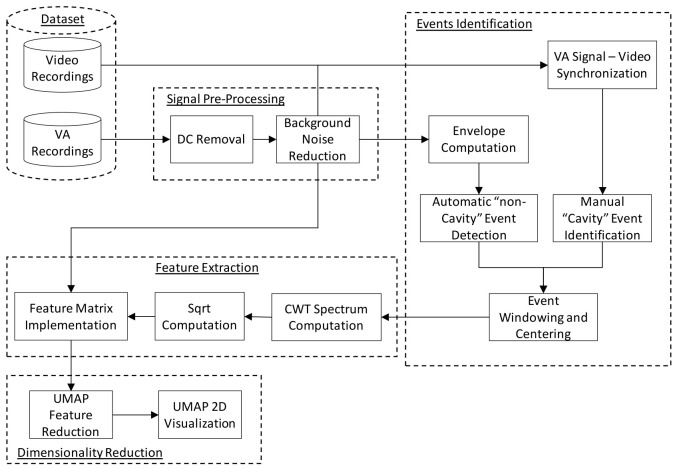
Overview of the data analysis workflow. The process has four stages: signal pre-processing, event identification, feature extraction, and dimensionality reduction. The diagram shows the steps from raw vibroacoustic signal acquisition and synchronization with video data, to identifying puncture-related events, extracting temporal and spectral features, and reducing the feature dimensions for visualization and clustering.

The workflow for vibroacoustic data analysis had four stages (see diagram in Figure 3): signal pre-processing, event identification, feature extraction, and dimensionality reduction.

#### 2.2.1. Signal Pre-Processing

The vibroacoustic signal was pre-processed to remove background noise and slow drifts. The goal was to keep only the signal components related to tool–tissue interactions.

First, the raw signal was centered by removing its DC offset. This reduced low-frequency drift and stabilized the baseline. Then, a spectral gating method was applied. The signal spectrogram was computed, and a frequency-dependent noise threshold was estimated. A binary mask suppressed the spectral components below this threshold. This reduced background noise while keeping short transient activity from needle–tissue interactions.

#### 2.2.2. Event Identification

The objective of the described study was to assess whether peritoneum punctures in human body donors can be identified by vibroacoustic signal analysis (see Section 1). In line with this objective, the events of interest were the peritoneal puncture and other detectable non-peritoneal events (such as skin or fascia punctures, collisions, or friction) during Veress needle insertion. The signal was annotated with two classes: the peritoneal puncture and all other non-peritoneal excitations in the vibroacoustic signal.

The event annotation framework was retrospective and anchored to the synchronized endoscopic and observation video. Specifically, the vibroacoustic signal was synchronized with the video by aligning their timelines so that the first vibroacoustic sample matched the first video frame. Synchronization was achieved by using the following reference points:

- Needle–surface contact: the moment the Veress needle enters in contact with the abdominal wall (visible in the observation video).
- Peritoneum puncture: the moment the needle crosses the peritoneal layer (visible in endoscopic video and confirmed from the view of the monitor in the external recording).

After synchronization, the annotated peritoneal puncture times of the endoscopic video were mapped directly to the vibroacoustic signal. We emphasise that this annotation pipeline is post-hoc and depends on an external optical reference; it does not by itself demonstrate prospective, video-independent detection. This dependency is addressed in Section 4.

To detect non-peritoneal events, the signal envelope was computed with the Hilbert transform for the segment starting at first skin contact and ending 200 ms before the annotated peritoneal puncture. A peak detector was applied to the envelope, with a minimum spacing of 20 ms between peaks.

This process resulted in two types of events:

1. Peritoneal puncture events identified in the video,
2. Automatically detected non-peritoneal interactions from the vibroacoustic signal.

Each event was centered at its maximum energy point, windowed in a 300 ms interval, and stored for later feature extraction.

#### 2.2.3. Feature Extraction

For each event segment, the Continuous Wavelet Transform (CWT) was calculated to capture the time–frequency structure of the signal. CWT was chosen instead of FFT-based methods because needle–tissue interactions result in short and non-stationary transient excitations. The Morse wavelet was used as the mother wavelet.

To reduce the influence of strong puncture events and highlight weaker dynamics, the square root of the CWT magnitude matrix was taken. The coefficients across all scales were then concatenated into a single feature vector to normalize the energies. The corresponding 300 ms time-domain segment was also appended so that each feature vector contained both, temporal and spectral information.

This produced a feature matrix in which each row represented one vibroacoustic event, and each column represented a feature derived from the CWT or time-domain waveform.

#### 2.2.4. Dimensionality Reduction

Dimensionality reduction was performed with Uniform Manifold Approximation and Projection (UMAP), a non-linear method that maps high-dimensional data into a lower-dimensional space while maintaining local and global structure [39]. UMAP is unsupervised, so the embedding reflects the natural structure of the data without using labels.

The feature matrix obtained during feature extraction (see Section 2.2.3) was used as input for UMAP. This allowed the model to use both the temporal and spectral properties of the events. The UMAP parameters were as follows:

- Number of neighbors: 4;
- Metric: cosine (chosen to emphasize relative spectral patterns rather than absolute magnitude);
- Minimum distance: 0.1;
- Spread: 0.1;
- Number of epochs: 1000.

UMAP has been used in biomedical and vibroacoustic signal analysis, where it has been shown to reveal a low-dimensional structure in complex feature spaces [40,41]. The resulting two-dimensional embedding provides a visual map of the feature space. The clusters on this map correspond to different types of vibroacoustic events. Peritoneal puncture events can be visually separated from other non-cavity interactions.

Two unsupervised clustering metrics were used to quantify cluster separation:

- Silhouette coefficient: measures how well the samples fit within their clusters (higher is better) [42].
- Davies–Bouldin index: measures the average similarity between clusters (lower is better) [43].

## 3. Results

The purpose of this study was to determine whether the vibroacoustic signals obtained during Veress needle insertion in a human body donor contain signal characteristics that serve to differentiate peritoneal punctures from other Veress needle events and thereby corroborate previous findings in animal cadavers. It was also examined whether the signal phases previously observed in porcine cadavers can be found in humans as well. In addition, it was assessed whether peritoneal punctures can be statistically distinguished based on their signal characteristics (see Section 1). This section reports the results for these objectives.

### 3.1. Identification of Peritoneum Punctures

A Veress needle with a proximal sensing unit was inserted into the abdomen of a single human body donor. 14 insertions were successful and included in the analysis.

**Figure 4:**
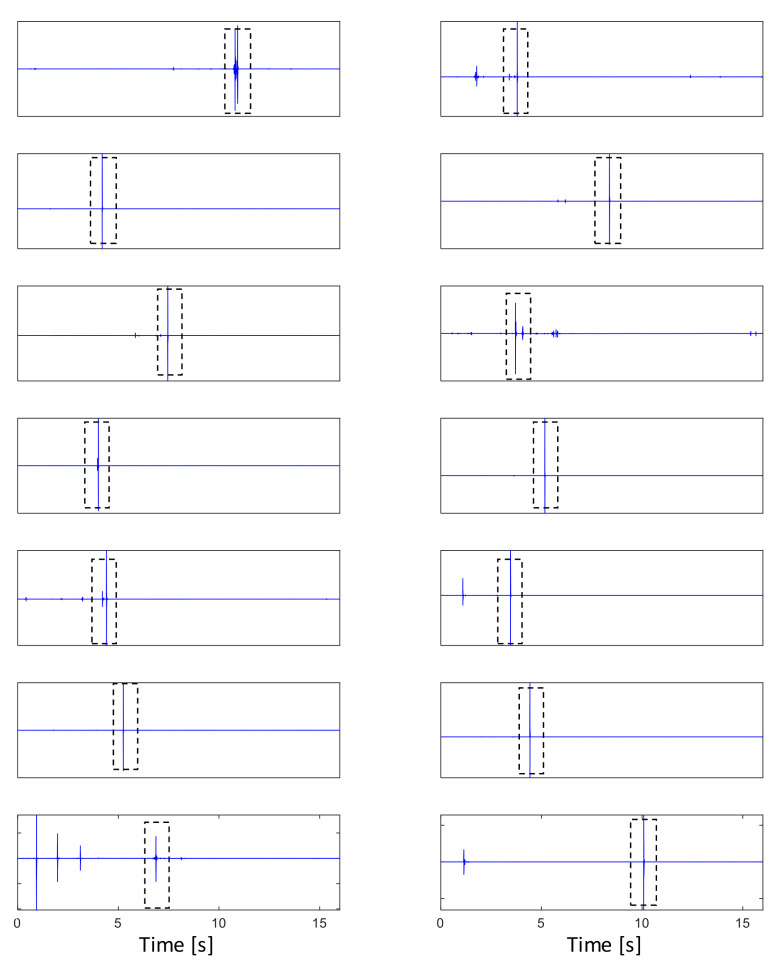
Vibroacoustic signals acquired from 14 Veress needle insertions in a human body donor. Each insertion contains a distinct transient excitation corresponding to the peritoneal cavity puncture (dashed boxes). Since the energy of the peritoneal puncture is markedly higher than the energy of other events such as friction, fascia passage or collisions, those events are rarely visible in this static plot.

Figure 4 qualitatively shows the vibroacoustic signals of all 14 insertions. Each signal covers the first 16 s of insertion, with the first sample representing the initial contact of the needle with the skin. In every insertion, the puncture to the peritoneal cavity produced a clear transient excitation, marked by dashed boxes. In 13 of 14 insertions (92.9%), the peritoneal puncture had a higher amplitude than all other interaction events, such as friction, fascia passage, or collision. Importantly, this comparison is performed within each insertion and does not yet establish a generalizable absolute threshold across insertions or subjects.

**Figure 5:**
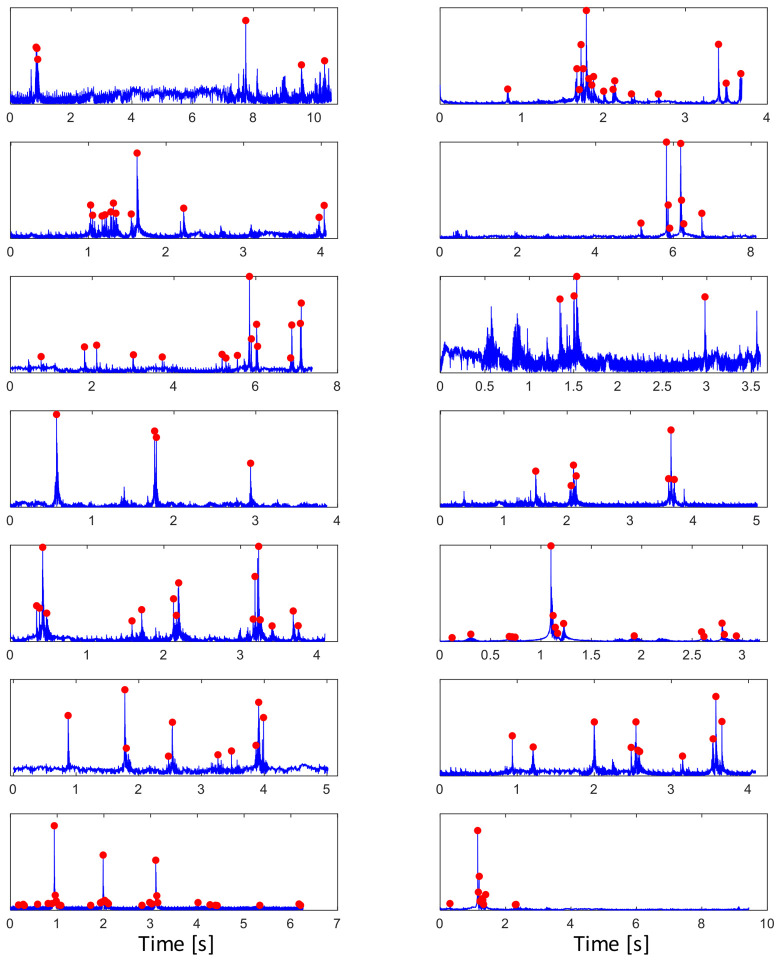
Envelope of the pre-processed vibroacoustic signal up to 200 ms before the peritoneal puncture. The plot shows automatically detected non-cavity events (red dots) appearing before the peritoneal puncture.

To compare other events to peritoneum puncture events, the envelope of the pre-processed signal was computed. Figure 5 shows each envelope from initial skin contact up to 200 ms before the peritoneal puncture. Several non-peritoneal excitations are visible, including friction events and punctures of the fascia or other tissues. Red dots mark automatically detected peak positions. Across all insertions, we identified 14 peritoneal puncture events (not visible in Figure 5) and 49 non-peritoneal events.

**Figure 6:**
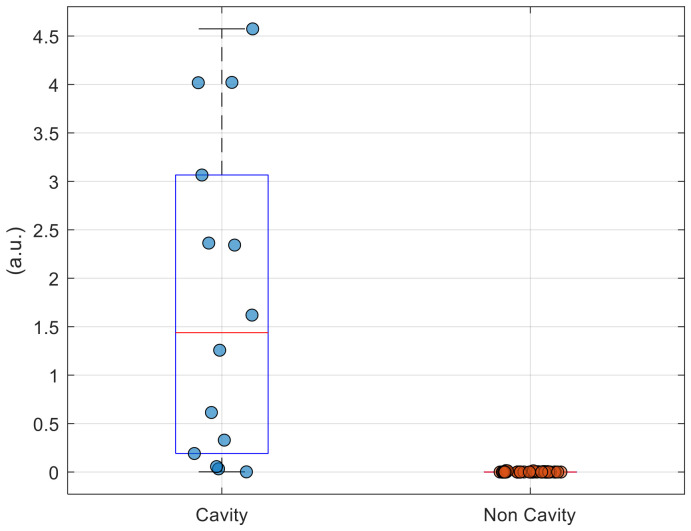
Energy comparison for cavity (blue) and non-cavity (red) events. Peritoneal punctures show higher energy content. This plot describes group-level differences and does not by itself establish an absolute decision threshold.

Figure 6 statistically compares the energy of peritoneal puncture events (displayed in Figure 4) and non-peritoneal events (displayed in Figure 5) using a box plot. Peritoneum punctures (blue) have clearly higher energy values than non-peritoneum events (red). This separation aligns with the annotated event classes and reflects the higher intensity of the cavity puncture compared to other event excitations. As emphasized in Section 4, this is a descriptive group-level comparison and does not by itself establish an absolute decision rule for individual events.

### 3.2. Observability of Characteristic Signal Phases

**Figure 7:**
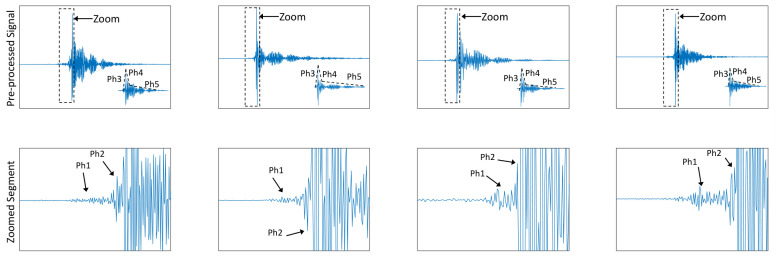
Time-domain vibroacoustic signals of four peritoneal puncture events in the human body donor. All characteristic phases (Ph1–Ph5) are observed.

To verify the findings of porcine cadavers, we examined the presence of characteristic phases associated with peritoneum puncture. Figure 7 shows four representative peritoneum puncture events from the human donor. The same temporal structure reported in [37] is observed.

Phases 3 to 5 are visible in all signals in relation to the needle mechanics:

- Phase 3 (Ph3): rapid release of spring tension,
- Phase 4 (Ph4): decline phase following release,
- Phase 5 (Ph5): release phase after spring relaxation.

By enlarging the segment before Ph3, the first two phases also become visible:

- Phase 1 (Ph1): friction between the inner and outer needle cores,
- Phase 2 (Ph2): rupture of the peritoneal membrane.

All five phases (Ph1–Ph5) appear in the human donor data, which corresponds to the dynamics found in porcine experiments. This shows that the vibroacoustic signature of peritoneal puncture is reproducible between both species under the experimental conditions of this study.

**Figure 8:**
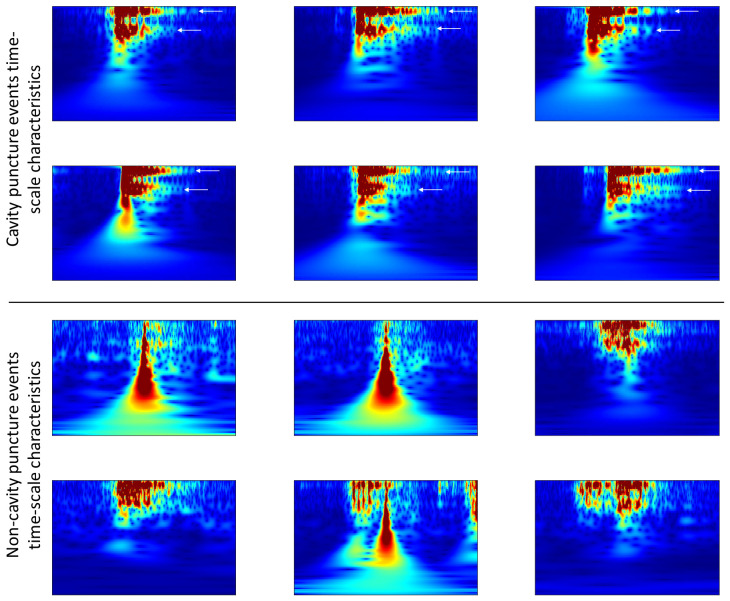
Continuous-Wavelet-Transformation (CWT) spectra of peritoneal puncture events (**top**) and non-cavity events (**bottom**). In the peritoneal puncture examples, red/yellow regions indicate high wavelet coefficient magnitudes, corresponding to broadband excitations occurring at cavity entry. The arrows highlight distinct spectral lines that emerge after puncture and are attributed to reduced damping of the needle tip within the cavity. In contrast, non-cavity events lack these persistent spectral-line patterns and predominantly exhibit transient broadband responses. spectra of peritoneal puncture events.

Figure 8 compares CWT spectra of six peritoneum punctures and six non-peritoneum events. Peritoneal punctures produce rapid, high-amplitude transients that appear as vertical broadband structures. A key difference is the reduced damping of the needle tip after peritoneum puncture: once the tip has crossed the peritoneum, it can oscillate more freely, producing sustained spectral lines immediately after the main excitation peak. These post-excitation spectral lines, marked with white arrows in Figure 8, appear only in peritoneum events. Their presence highlights a clear spectral distinction between cavity entry and other interactions under the present experimental conditions.

### 3.3. Statistical Differentiation Between Peritoneum and Non-Peritoneum Punctures

**Figure 9:**
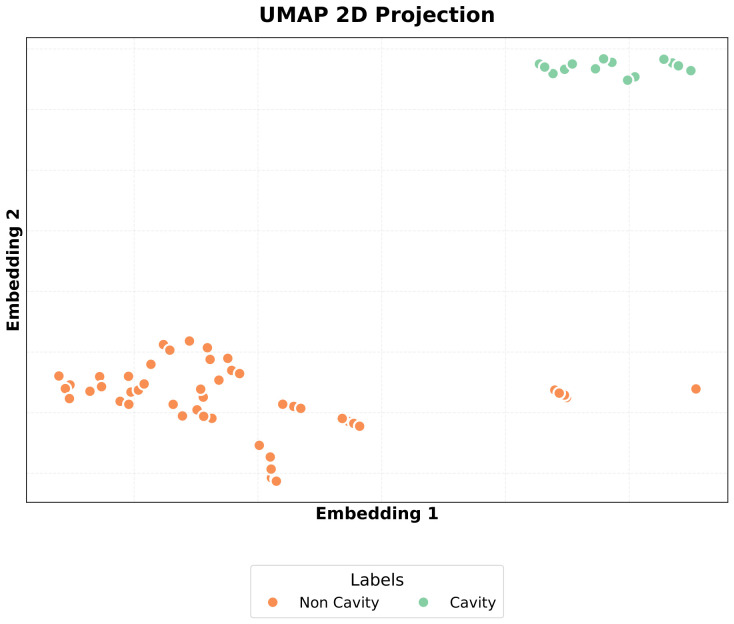
UMAP projection of vibroacoustic events showing separation between peritoneum (green) and non-peritoneum (orange) events.

Figure 9 shows the two-dimensional UMAP projection of all detected events. Non-peritoneum events are shown in orange; peritoneum events are shown in green. The projection shows a clear separation between the two classes. The embedding was computed using the combined feature matrix of pre-processed time-domain segments and CWT–sqrt features. Peritoneum events form a compact cluster with consistent characteristics, while non-peritoneum events appear more spread out due to greater variability. Thus, the qualitative observations in time and time-frequency are confirmed by the structure of the feature space.

A calculation of the Davies–Bouldin Index (DBI) and the Silhouette Score for the UMAP projection yielded a DBI value of 0.233 and a Silhouette score of 0.793. While the DBI value indicates compact clusters with strong separation, the Silhouette Score confirms a clear distinction between event types.

Both metrics support the visual clustering observed in Figure 9. The combined temporal and spectral features provide a stable, unsupervised characterization of signal dynamics and capture meaningful differences between peritoneal punctures and other events. We note that these metrics describe the geometry of an unsupervised embedding; they characterize separability of the two event classes in the feature space but should not be read as clinical performance estimates such as sensitivity or specificity for prospective in vivo use.

## 4. Discussion

This pilot study demonstrates that, under the controlled ex vivo conditions of a single human body donor, proximal vibroacoustic sensing was feasible for detecting peritoneal puncture during Veress needle insertion. The distinct signal phases (Ph1–Ph5) and transient excitation patterns previously identified in porcine cadavers were consistently reproduced in human tissue, including comparable temporal profiles and relative energy distributions. To the best of our knowledge, this is the first time the proximal vibroacoustic sensing concept has been evaluated on a human body donor and the first cross-species replication of the previously reported puncture phase structure on human tissue. The contribution of this work is therefore twofold. First, it establishes a translational stepping stone between previous porcine-cadaver studies and the in vivo investigations that are ultimately required for clinical deployment: the same instrument, signal-acquisition prototype, and analysis pipeline used in animal experiments are now shown to operate on human anatomy without modification. Second, it provides the first description of the cross-species robustness of the characteristic vibroacoustic phases of peritoneal entry, supporting the hypothesis that these phases reflect intrinsic mechanics of the needle-tissue system rather than species-specific tissue properties. Together, these findings strengthen the case for vibroacoustic sensing as a non-invasive, add-on guidance modality compatible with standard Veress needles and existing surgical workflows.

A central methodological consideration for the present study concerns the abdominal-cavity conditions at the moment of peritoneal entry. As described in Section 2, no formal pneumoperitoneum was established. The cadaver’s abdominal wall had collapsed post-mortem, and an experienced laparoscopic surgeon had to apply slight pressure to the abdominal cavity so that the endoscopic camera could provide a usable view; the pressure generated in this way was significantly lower than the standard clinical insufflation pressure of 10–12 mmHg. Two consequences follow. On the one hand, the experimental conditions are closer to the clinical situation than they would have been under full pneumoperitoneum, because no externally insufflated gas cushion was present at the moment of needle entry. On the other hand, even this small amount of applied pressure may slightly alter abdominal wall tension and the mechanical loading on the needle tip after peritoneal entry, and could in principle modify both the puncture transient and the post-puncture oscillations visible in Figure 8. The magnitude of this effect cannot be quantified within the present single-donor design and should be assessed in future studies that systematically vary the applied intra-abdominal pressure (including the clinically realistic 0 mmHg condition).

A second translational caveat is the use of a fresh human body donor rather than a living patient. Despite measurements being performed within 48 h post-mortem, post-mortem tissue differs from living tissue in several mechanically and acoustically relevant ways: there is no perfusion or active muscle tone, tissue elasticity and water content evolve progressively after death, and respiratory and cardiac motion are absent. These differences may alter both the damping experienced by the needle along its path and the transient excitation generated at peritoneal entry. The consistency we observe between the present cadaveric data and the previous porcine cadaveric data is therefore informative about the robustness of the vibroacoustic signature across species and individuals, but it does not by itself guarantee that the same signature will be present, or equally distinguishable, in living patients. In vivo evaluation, including in patients with varying BMI, abdominal wall thickness, prior surgery, and pathological conditions, is required.

Peritoneal events form a compact cluster in the combined time-and-spectral feature space, with a Davies–Bouldin Index of 0.233 and a Silhouette score of 0.793, and the peritoneal puncture is the highest-energy event of its insertion in 13 of 14 cases. These analyses indicate that an event-level decision rule is plausible, but they do not yet demonstrate prospective, video-independent detection: the peritoneal-puncture annotations themselves were derived from synchronized endoscopic video, and a generalizable absolute threshold across subjects cannot be established from a single donor. Establishing such a rule, together with sensitivity and specificity estimates from prospective data, is identified as a priority for follow-up work.

Differentiating peritoneal entry from non-peritoneal events is clinically important. Misplacement of the Veress needle-such as pre-peritoneal positioning or unintended organ injury-remains a major source of complications in laparoscopic surgery. Conventional tactile cues and the Veress needle’s spring mechanism are frequently criticized for their subjectivity, particularly among less experienced surgeons. In this study, peritoneal punctures exhibited signal characteristics distinct from other tissue transitions in both the time-domain phase structure (Figure 7) and the time–frequency response (Figure 8), and the two event classes were separable in the unsupervised feature embedding (Figure 9). This supports the broader hypothesis that vibroacoustic signals carry enough information to ground an automated, surgeon-independent indication of peritoneal entry. The 14 insertions were distributed across three standard primary access points (subumbilical region, Lee–Huang point, Palmer’s point) as summarized in Table 1. Within these sites, individual punctures were spatially separated by a minimum of approximately 2 cm and adjacent sites were not punctured in immediate succession in order to minimize procedural artifacts from neighboring previous insertions. Nevertheless, repeated punctures of the same abdominal wall in a single donor cannot be considered fully independent observations, and tissue-level changes from preceding insertions may have influenced subsequent signals.

The most informative next steps are (i) a multi-donor study with controlled variation of intra-abdominal pressure, (ii) an in vivo evaluation in a porcine model and subsequently in patients, with a pre-specified prospective decision rule and without an external optical reference, and (iii) extension of the event vocabulary beyond peritoneal entry to clinically relevant adverse events such as bowel or vascular contact. Set against the existing literature, the principal value of the present study is that it is the first demonstration of proximal vibroacoustic sensing on a human body donor and the first confirmation that the puncture phase structure described in porcine cadavers transfers to human anatomy. This significantly narrows the translational gap between previous animal-cadaver work and future in vivo studies, and supports the case for advancing vibroacoustic sensing toward clinical trials in laparoscopic access.

## 5. Conclusions

This pilot study provides the first single-donor evidence that proximal vibroacoustic sensing can capture meaningful access-related signal transitions during Veress needle insertion in human tissue, and that the characteristic signal phases previously described in porcine cadavers transfer to human anatomy. To our knowledge, this is the first evaluation of the proximal vibroacoustic sensing concept on a human body donor and therefore an important translational step between earlier animal-cadaver work and future in vivo investigations. The peritoneal puncture was the highest-energy event of its insertion in 13 of 14 cases, and peritoneal events formed a compact, well-separated cluster in the unsupervised feature embedding, supporting the case for vibroacoustic sensing as a non-invasive, add-on guidance modality that integrates with standard Veress needles and existing surgical workflows.

These results should, however, be interpreted within the limits of the experimental design. The use of a single fresh body donor, a single experienced operator, and a retrospective video-anchored annotation framework, mean that the present work demonstrates feasibility and translational replication rather than being an indicator of the techniques clinical performance. Continued validation across multiple donors, multiple operators, and ultimately in vivo studies with prospective, video-independent decision rules will be necessary to determine whether vibroacoustic sensing is suitable for real-time decision support in laparoscopic surgery.

## Figures and Tables

**Table 1:** Distribution of the 14 Veress needle insertion sites across the standard primary access points used in this study. Site assignments were guided by the recommended primary entry points described in [38].

Anatomical Location	Number of Insertions	Approximate Location
Subumbilical region	5	Midline, immediately inferior to umbilicus
Lee–Huang point	4	Upper midline, between xiphoid and umbilicus
Palmer’s point (LUQ)	5	Left upper quadrant, ∼3 cm below costal margin
Total	14	

## Data Availability

The data presented in this study are available on request from the corresponding author due to specific regulations and policies within the organizations involved in this study.
